# Flash Communication: An *ortho*-Trifluoromethylphenyl Substituted Phosphine Ligand for Applications in Gold(I) Catalysis

**DOI:** 10.1021/acs.organomet.5c00240

**Published:** 2025-08-18

**Authors:** Itxaso Bustos, Nil Roig, Adrian B. Chaplin

**Affiliations:** † Department of Chemistry, 2707 University of Warwick, Coventry CV4 7AL, U.K.; ‡ Facultad de Química de San Sebastián, Universidad del País Vasco (UPV/EHU), Apdo. 1072, 20080 San Sebastián, Spain; § Eenheid Algemene Chemie (ALGC), Vrije Universiteit Brussel (VUB), 1050 Brussels, Belgium

## Abstract

Synthesis of the new bulky phosphine ligand di­(1‑adamantyl)-2-trifluoromethyphenylphosphine is reported, which coordinates with the trifluoromethyl group projected toward the metal center and exhibits a %*V*_bur_ of 47.3%. Well-defined gold­(I) derivatives demonstrate higher catalytic activity for the cycloisomerization of 4-fluoro-*N*-(prop-2-yn-1-yl)­benzamide than di­(1‑adamantyl)-2-biphenylphosphine (AdJohnPhos) and di­(1‑adamantyl)­phenylphosphine analogues.

## Graphical abstract

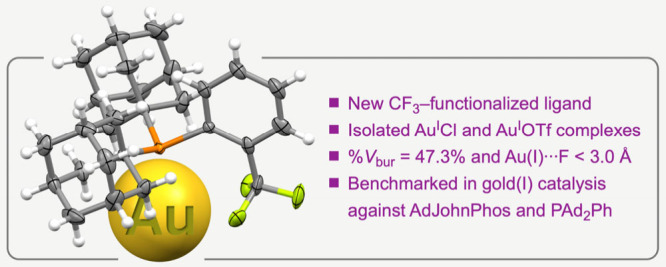

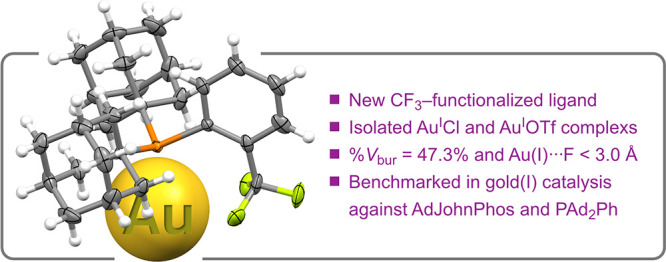

Homogeneous gold catalysis is a powerful synthetic tool, which enables the efficient preparation of a wide variety of organic molecules and natural products.^1^ The electrophilic activation of alkynes by monoligated gold­(I) cations, in particular, is a widely exploited strategy for the construction of carbon–carbon and carbon–heteroatom bonds.^2^ Tuning the reactivity and stability of these catalytically active low coordinate gold­(I) fragments is key to effective catalysis and is typically realized by selection of a bulky phosphine or *N*-heterocyclic carbene ancillary ligand, in combination with a weakly coordinating anion and solvent.^3,4^ In this context, gold­(I) complexes of biaryl-substituted, Buchwald-type, phosphine ligands are often the precatalysts of choice with the sterically imposing profile of these ligands conferred by projection of the *ortho*-aryl group into the coordination sphere of the metal (Figure 1A).^3,5^ As part of our work exploring the coordination chemistry of *ortho*-trifluoromethylphenyl substituted phosphine ligands,^6^ we speculated that this bulky, electron-deficient P-substituent could be beneficial in electrophilic gold­(I) catalysis. We herein present our preliminary findings evaluating this hypothesis using conformationally rigid phosphine ligand **L1** (Figure 1B), in which the weakly interacting trifluoromethyl appendage is directed at the metal binding site.

**Figure 1:**
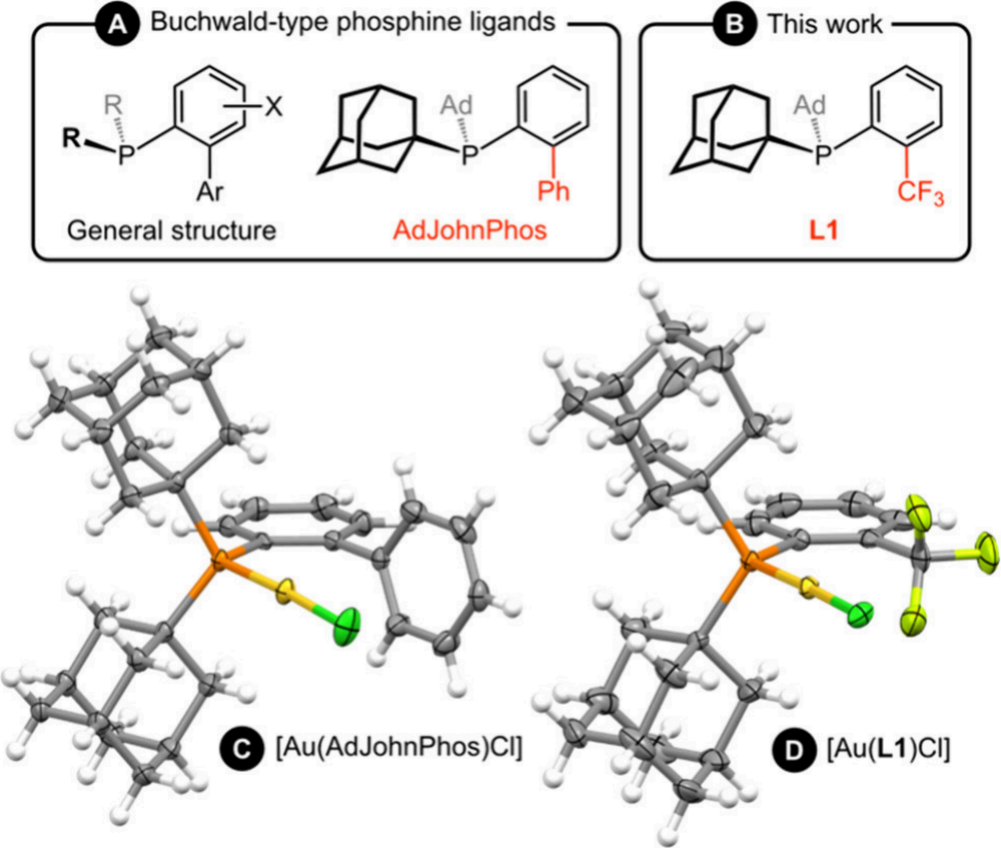
Buchwald-type phosphine ligands (**A**), the *ortho*-trifluoromethylphenyl substituted phosphine ligand **L1** (**B**), and solid-state structures of gold­(I) chloride complexes of AdJohnPhos (**C**, CCDC 1550231) and **L1** (**D**) with thermal ellipsoids at 50%.

The new phosphine ligand **L1** was prepared following an adapted literature procedure, involving palladium-catalyzed coupling of PAd_2_H and 2-bromobenzotrifluoride,^7^ and isolated in 53% yield. The gold­(I) chloride complex was thereafter obtained by reaction with [Au­(SMe_2_)­Cl], isolated in 78% yield, and fully characterized. Coordination of the phosphine is marked by a large downfield shift of the quartet ^31^P resonance δ 24.9 → 96.4 and reduction in the ^TS^ *J*_PF_ coupling constant (57 → 14 Hz) but only a minor perturbation to the corresponding ^19^F resonance δ –54.3 → –57.0. The solid-state structure demonstrates a close approach of the CF_3_ appendage to the gold­(I) center, which is straddled by two C–F bonds with Au···F contacts of 2.986(6) and 2.988(7) Å (Figure 1D). Crystallographically characterized instances of noble metal M···F_3_C bonding are scarce and, using Plenio’s distance threshold of 3.0 Å for a significant M···F interaction,^8^ limited to a handful of silver-cation-based systems, epitomized by Ag­[Al­(OC­(CF_3_)_3_)_4_][Bibr ref9] and group 9 complexes from our group (M···F = 2.36–2.54 Å; CSD v5.46).^6^ Further analysis using the SambVca2 program enabled the steric profile of **L1** to be quantified,^10^ with the resulting %*V*_bur_ value of 47.3% only slightly smaller than that measured for AdJohnPhos (51.4%) from the known gold­(I) chloride derivative (Figure 1C).^11^

The cycloisomerization of *N*-propargyl benzamides is a useful model reaction for assessing the relative performance of homogeneous gold­(I) catalysts.^12^ The transformation of 4-fluoro-*N*-(prop-2-yn-1-yl)­benzamide can be conveniently followed by ^19^F NMR spectroscopy and was selected to benchmark the utility of **L1** in catalysis relative to AdJohnPhos (Scheme 1).^13^ Under the mild conditions used, [Au­(**L1**)­Cl] proved to be an active precatalyst, in combination with the halide abstracting agent Na­[BAr^F^_4_] (Ar^F^ = 3,5-(CF_3_)_2_C_6_H_3_),^14^ delivering the 5-*exo*-cyclization product with an initial TOF of ∼ 40 h^–1^: over three times greater than the Buchwald system. Complete consumption of the substrate was observed within 6 h (1 mol % catalyst loading), and comparable catalytic activity was observed when the isolated “preactivated” triflate derivative [Au­(**L1**)­(OTf)] was used instead. Emphasizing the decisive role of ligand sterics in this reaction, low catalytic activity was observed when using PAd_2_Ph (%*V*_bur_ = 37.6%) as the ancillary ligand.

**Scheme 1:**
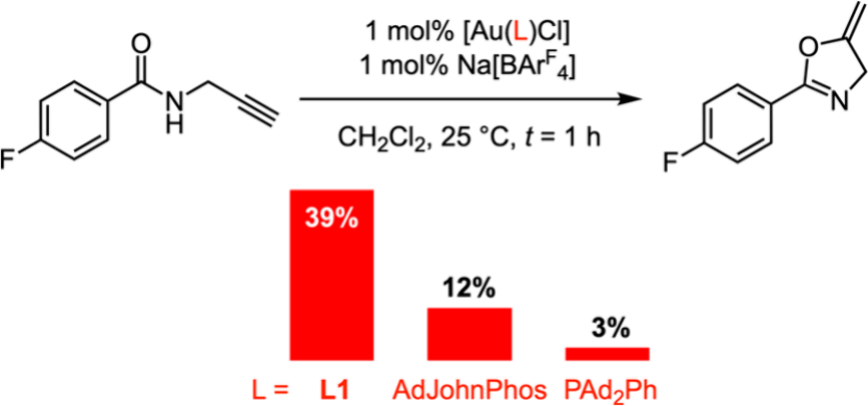
Gold­(I) Catalyzed Cycloisomerization of 4-Fluoro-*N*-(prop-2-yn-1-yl)­benzamide^a^

a Conditions: 1.5 mM of precatalyst, 1.5 mM of Na­[BAr^F^_4_], and 0.15 M substrate in CH_2_Cl_2_ stirred under argon. Conversion determined by analysis of aliquots diluted with CH_3_CN using ^19^F NMR spectroscopy.

We hope these findings stimulate further investigation into the late transition metal coordination chemistry of **L1** and its applications in homogeneous catalysis.

## Supplementary Material



## Notes

Full experimental details and characterization data for new complexes and selected reactions (PDF)

## Notes

Deposition Numbers 2467804–2467807 contain the supplementary crystallographic data for this paper. These data can be obtained free of charge via the joint Cambridge Crystallographic Data Centre (CCDC) and Fachinformationszentrum Karlsruhe Access Structures service.

## References

[1] Hashmi A. S. K. (2021). Introduction: Gold Chemistry. Chem. Rev..

[2] Dorel R., Echavarren A. M. (2015). Gold­(I)-Catalyzed
Activation of Alkynes
for the Construction of Molecular Complexity. Chem. Rev..

[3] Collado A., Nelson D. J., Nolan S. P. (2021). Optimizing
Catalyst
and Reaction Conditions in Gold­(I) Catalysis – Ligand Development. Chem. Rev..

[4] Lu Z., Li T., Mudshinge S. R., Xu B., Hammond G. B. (2021). Optimization of
Catalysts and Conditions in Gold­(I) Catalysis – Counterion
and Additive Effects. Chem. Rev..

[5] Zuccarello G., Zanini M., Echavarren A. M. (2020). Buchwald-Type
Ligands on Gold­(I) Catalysis. Isr. J. Chem..

[6] Poole E. W., Bustos I., Hood T. M., Smart J. E., Chaplin A. B. (2023). Iridium Complexes of an *ortho*-Trifluoromethylphenyl
Substituted PONOP Pincer Ligand. Dalton Trans..

[7] Hu H., Wang Y., Qian D., Zhang Z.-M., Liu L., Zhang J. (2016). Enantioselective Gold-Catalyzed Intermolecular [2+2]-Cycloadditions
of 3-Styrylindoles with *N*-Allenyl Oxazolidinone. Org. Chem. Front..

[8] Plenio H. (1997). The Coordination
Chemistry of the CF Unit in Fluorocarbons. Chem.
Rev..

[9] Krossing I. (2001). The Facile
Preparation of Weakly Coordinating Anions: Structure and Characterisation
of Silverpolyfluoroalkoxyaluminates AgAl­(OR^F^)_4_, Calculation of the Alkoxide Ion Affinity. Chem.—Eur. J..

[10] Falivene L., Credendino R., Poater A., Petta A., Serra L., Oliva R., Scarano V., Cavallo L. (2016). SambVca 2. A Web Tool
for Analyzing Catalytic Pockets with Topographic Steric Maps. Organometallics.

[11] Rotta-Loria N. L., Chisholm A. J., MacQueen P. M., McDonald R., Ferguson M. J., Stradiotto M. (2017). Exploring the Influence of Phosphine
Ligation on the
Gold-Catalyzed Hydrohydrazination of Terminal Alkynes at Room Temperature. Organometallics.

[12] Yu C., Hsiao Y., Löffler J., Kaiser N., Huang B., Lee C., Hung C., Shen J., Yap G. P. A., Gessner V. H., Ong T. (2025). Increasing
the Donor Strength of Alkenylphosphines by Twisting the
C=C Double Bond. Angew. Chem., Int. Ed..

[13] Wilkins L. C., Kim Y., Litle E. D., Gabbaï F. P. (2019). Stabilized Carbenium Ions as Latent,
Z-type Ligands. Angew. Chem., Int. Ed..

[14] No turnover was observed in the absence of Na[BAr^F^ _4_].

