# Supplementary material for: Flash Communication: An ortho-Trifluoromethylphenyl Substituted Phosphine Ligand for Applications in Gold(I) Catalysis
Source: Organometallics. 2025 Aug 18;44(17):1881–3. doi: 10.1021/acs.organomet.5c00240 (PMC12422020; doi:10.1021/acs.organomet.5c00240)
Supplement: Supplementary file 1 [file om5c00240_si_001.pdf]

Supporting information for:

Flash Communication: An *ortho*-Trifluoromethylphenyl Substituted Phosphine Ligand for Applications in Gold(I) Catalysis

Itxaso Bustos,[a,b] Nil Roig,[a,c] and Adrian B. Chaplin[a,*]

[a] *Department of Chemistry, University of Warwick, Coventry CV4 7AL, U.K.;*
E-mail: a.b.chaplin@warwick.ac.uk
[b] *Facultad de Química de San Sebastián, Universidad del País Vasco (UPV/EHU), Apdo. 1072, 20080 San Sebastián, Spain*
[c] *Eenheid Algemene Chemie (ALGC), Vrije Universiteit Brussel (VUB), 1050 Brussels, Belgium*

Table of contents

1 General methods S1

2 Preparation of PAd_2(2-trifluoromethylphenyl) **L1** S2

3 Preparation of [Au(**L1**)Cl] S5

4 Preparation of [Au(**L1**)(OTf)] S8

5 Steric profile of AdJohnPhos S11

6 Preparation of [Au(PAd_2Ph)Cl] S12

7 Catalysis data S15

8 References S16

1 General methods

All manipulations were performed under an atmosphere of argon using Schlenk and glove box techniques unless otherwise stated. Glassware was oven dried at 150 °C overnight and flame-dried under vacuum prior to use. Molecular sieves were activated by heating at 300 °C *in vacuo* overnight. CD_2Cl_2 was freeze-pump-thaw degassed and dried over activated 3 Å molecular sieves. Anhydrous toluene, CH_2Cl_2, diethyl ether, hexane, and pentane were purchased from Acros Organics or Sigma-Aldrich, freeze-pump-thaw degassed and stored over activated 3 Å molecular sieves. [Au(AdJohnPhos)Cl], [1] PAd_2Ph, [2] 4-fluoro-*N*-(prop-2-yn-1-yl)benzamide, [3] and Na[BAr^F_4] [4] were synthesized according to published procedures, or minor variations thereof. All other reagents are commercial products and were used as received. NMR spectra were recorded on Bruker

spectrometers under an argon atmosphere in J. Young valve NMR tubes at 298 K unless otherwise stated. Chemical shifts are quoted in ppm and coupling constants in Hz. NMR spectra in non-deuterated solvents were recorded using an internal capillary of C_6D_6. Crystallographic data were collected on a Rigaku Oxford Diffraction SuperNova AtlasS2 CCD using Mo Kα or Cu Kα radiation and an Oxford Cryosystems N-HeliX cryostat (150 K). Data were collected and reduced using CrysAlisPro. The structures were solved using SHELXT and refined using SHELXL, through the Olex2 interface.[5,6] All non-hydrogen atoms were refined anisotropically. Hydrogen atoms were placed in calculated positions and refined using the riding model. Full details for all structures reported are documented in the CIF, which have been deposited with the Cambridge Crystallographic Data Centre under CCDC 2467804–2467807. High resolution (HR) ESI-MS were recorded on Bruker Maxis Plus instrument and microanalyses were performed in duplicate at Elemental Microanalysis Ltd.

2 Preparation of PAd_2(2-trifluoromethylphenyl) L1

A solution of 1,1'-bis(diphenylphosphino)ferrocene (dppf, 456.8 mg, 82.4 μmol) in toluene (10 mL) was added to a flask charged with $HPAd_2$ (1.0 g, 3.4 mmol), $Pd(OAc)_2$ (154.4 mg, 68.8 μmol) and NaOMe (185.8 mg, 3.4 mmol). Additional toluene (120 mL) was added, followed by 1-bromo-2-(trifluoromethyl)benzene (928.8 mg, 4.1 mmol), and the reaction heated at reflux for 15 days. The solution was filtered through an 8 cm silica plug and reduced to dryness *in vacuo*. The product was obtained as colourless crystals by recrystallisation from hexane at room temperature. Yield: 808 mg (1.81 mmol, 53%). Single crystals suitable for X-ray diffraction were obtained in this manner.

^{1}H NMR (CD_2Cl_2, 500 MHz): δ 8.00 (d, $^2J_{HH}$ = 7.4, 1H, 6-Ar), 7.76 (ddd, $^3J_{HH}$ = 7.7, $^4J_{PH}$ = 3.3, $^4J_{HH}$ = 1.5, 1H, 3-Ar), 7.54 (br t, $^3J_{HH}$ = 7.4, 1H, 5-Ar), 7.49 (br t, $^3J_{HH}$ = 7.7, 1H, 4-Ar), 2.00 (d, $^2J_{HH}$ = 12.1, 6H, Ad{2-CH_2}), 1.89 (s, 6H, Ad{3-CH}), 1.84 (d, $^2J_{HH}$ = 12.1, 6H, Ad{2-CH_2}), 1.67 (unresolved AB, 12H, Ad{4-CH_2}).
^{13}C{^{1}H} NMR (CD_2Cl_2, 126 MHz): δ 138.7 (d, $^2J_{PC}$ = 1, 6-Ar), 137.8 (app pent, J = 28, 2-Ar), 135.2 (d, $^1J_{PC}$ = 42, 1-Ar), 129.9 (s, 5-Ar), 129.1 (s, 4-Ar), 126.6 (app pent, J = 7, 3-Ar), 124.8 (q, $^1J_{FC}$ = 275, CF_3), 42.2 (d, $^2J_{PC}$ = 13, Ad{2-CH_2}), 37.6 (d, $^1J_{PC}$ = 25, Ad{1-C}), 37.2 (s, Ad{4-CH_2}), 29.4 (d, $^3J_{PC}$ = 9, Ad{3-CH}).
^{19}F{^{1}H} NMR (CD_2Cl_2, 377 MHz): δ −54.3 (d, $^{TS}J_{PF}$ = 57).
^{31}P{^{1}H} NMR (CD_2Cl_2, 162 MHz): δ 24.9 (q, $^{TS}J_{PF}$ = 57).
HR ESI-MS (positive ion, 4 kV): 447.2417 ([M+H]$^+$, calcd 447.2423) *m/z*.
Anal. calcd for $C_{27}H_{34}F_3P$ (446.54 g·mol^{-1}): C, 72.63; H, 7.67; N, 0.00. Found: C, 72.14; H, 7.74; N, 0.00.

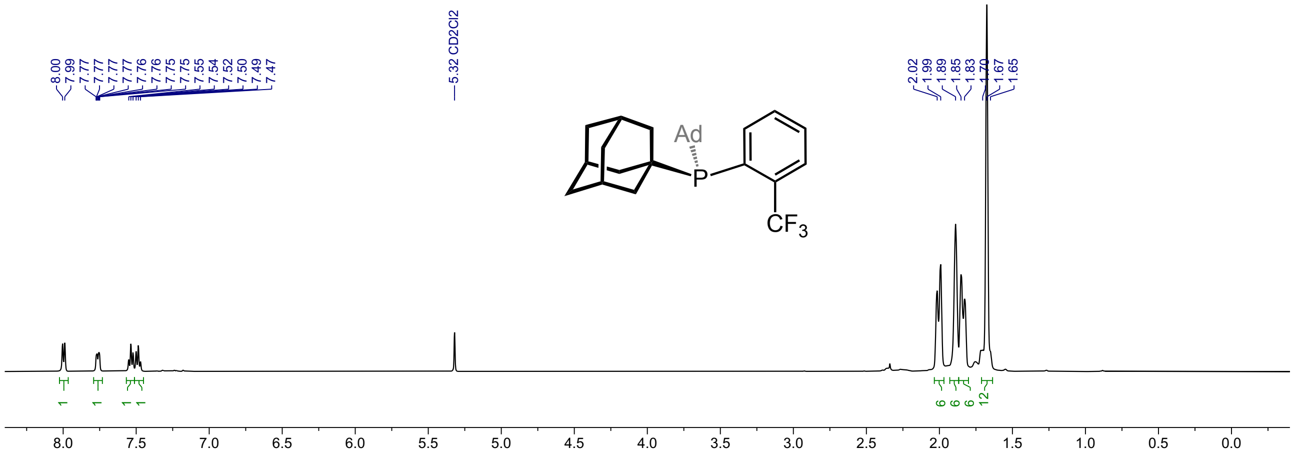

Figure S1. ^{1}H NMR spectrum of **L1** in CD_2Cl_2 (500 MHz).

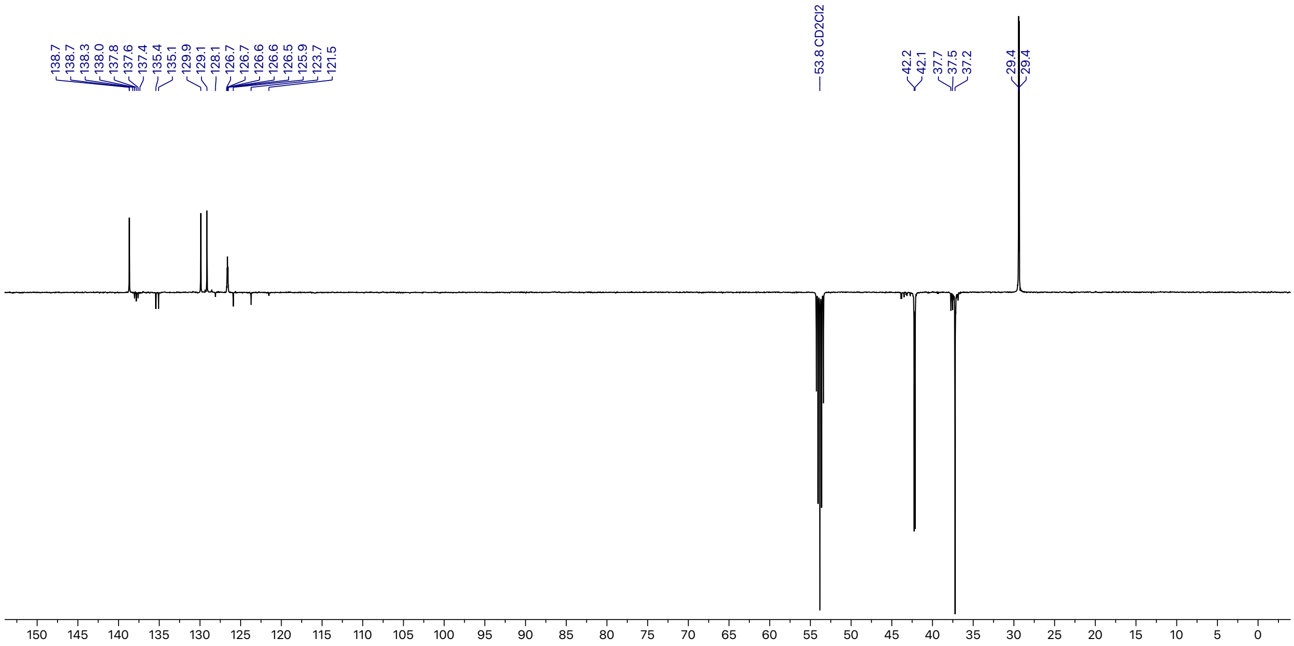

Figure S2. $^{13}C\{^{1}H\}$ APT NMR spectrum of **L1** in CD_2Cl_2 (126 MHz).

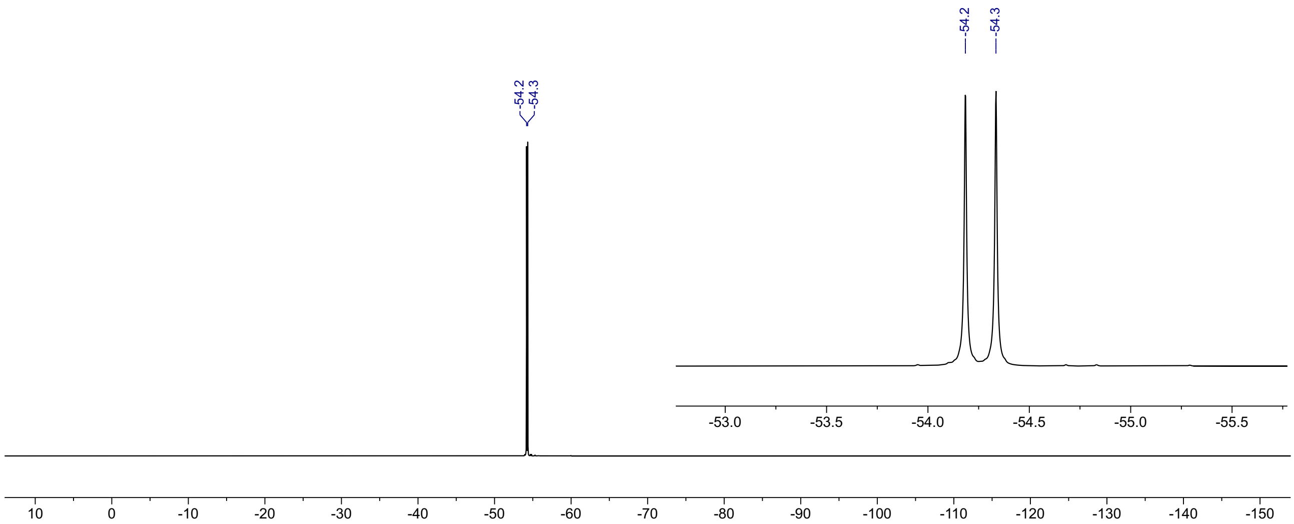

Figure S3. $^{19}F\{^{1}H\}$ NMR spectrum of **L1** in CD_2Cl_2 (377 MHz).

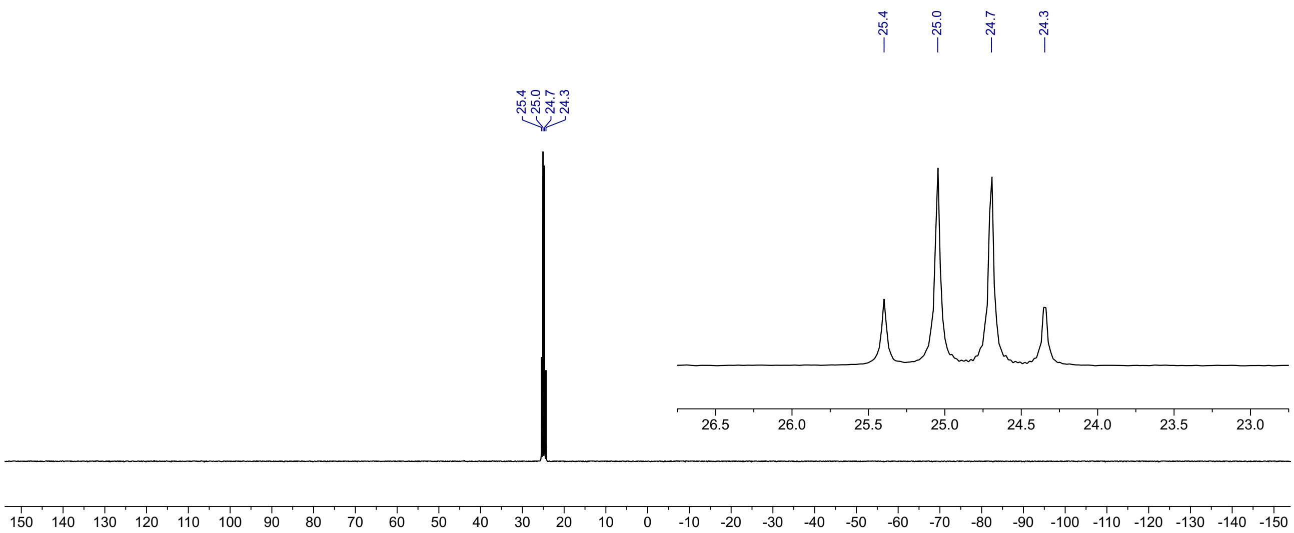

Figure S4. $^{31}P\{^{1}H\}$ NMR spectrum of **L1** in CD_2Cl_2 (162 MHz).

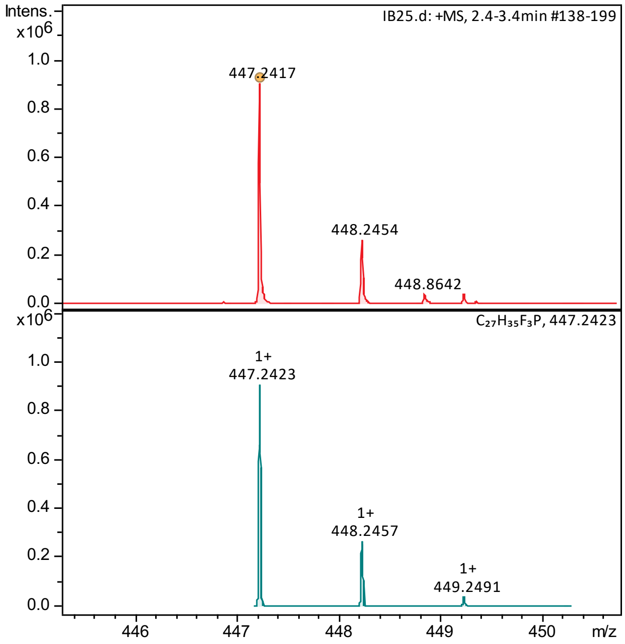

Figure S5. HR ESI-MS of **L1**.

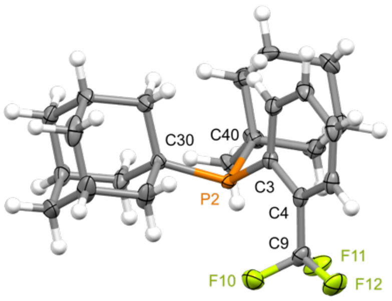

Figure S6. Solid-state structure of **L1** with thermal ellipsoids at 50%. Selected angles: P2–C3–C4, 120.40(9)°; C3–C4–C9, 122.11(10)°; C30–P2–C3–C4, 119.54(9)°; C40–P2–C3–C4, –123.22(9)°; C3–C4–C9–F12, 177.12(11)°.

3 Preparation of [Au(L1)Cl]

A solution of **L1** (151.5 mg, 340.0 μmol) and [Au(SMe$_2$)Cl] (100.0 mg, 340.0 μmol) in CH_2Cl_2 (10 mL) was stirred at room temperature for 16 h. Volatiles were removed *in vacuo* and the residue washed with diethyl ether (2×5 mL) to afford the product as a white solid. Yield: 179.0 mg (263.6 μmol, 78%). Single crystals suitable for X-ray diffraction were obtained by recrystallisation from CH_2Cl_2/ether at room temperature.

^{1}H NMR (CD_2Cl_2, 500 MHz): δ 7.99–8.05 (m, 1H, 6-Ar), 7.94–7.99 (m, 1H, 3-Ar), 7.68–7.74 (m, 2H, 4-Ar+5-Ar), 2.24 (dm, $^2J_{HH}$ = 11.1, 6H, Ad{2-CH$_2$}), 2.18 (dm, $^2J_{HH}$ = 11.1, 6H, Ad{2-CH$_2$}), 2.02 (br, 6H, Ad{3-CH}), 1.69 (unresolved AB, 12H, Ad{4-CH$_2$}).

^{13}C{^{1}H} NMR (CD_2Cl_2, 126 MHz): δ 137.6 (s, 6-Ar), 136.2 (qd, $^2J_{FC}$ = 30, $^2J_{PC}$ = 7, 2-Ar), 131.8 (d, $^4J_{PC}$ = 2, 4-Ar), 130.6 (d, $^3J_{PC}$ = 6, 5-Ar), 128.7 (app pent, J = 7, 3-Ar), 125.6 (d, $^1J_{PC}$ = 30, 1-Ar), 123.8 (q, $^1J_{FC}$ = 275, CF_3), 44.5 (d, $^1J_{PC}$ = 21, Ad{1-C}), 42.8 (d, $^2J_{PC}$ = 3, Ad{2-CH$_2$}), 36.6 (d, $^4J_{PC}$ = 1, Ad{4-CH$_2$}), 29.3 (d, $^3J_{PC}$ = 10, Ad{3-CH}).

^{19}F{^{1}H} NMR (CD_2Cl_2, 377 MHz): δ −50.1 (d, J_{PF} = 14).

^{31}P{^{1}H} NMR (CD_2Cl_2, 162 MHz): δ 67.9 (q, J_{PF} = 14).

HR ESI-MS (positive ion, 4 kV): 701.1604 ([M+Na]$^+$, calcd 701.1597) *m/z*.

Anal. calcd for $C_{27}H_{34}F_3PAuCl$ (678.96 g·mol^{-1}): C, 47.76; H, 5.05; N, 0.00. Found: C, 47.67; H, 4.93; N, 0.00.

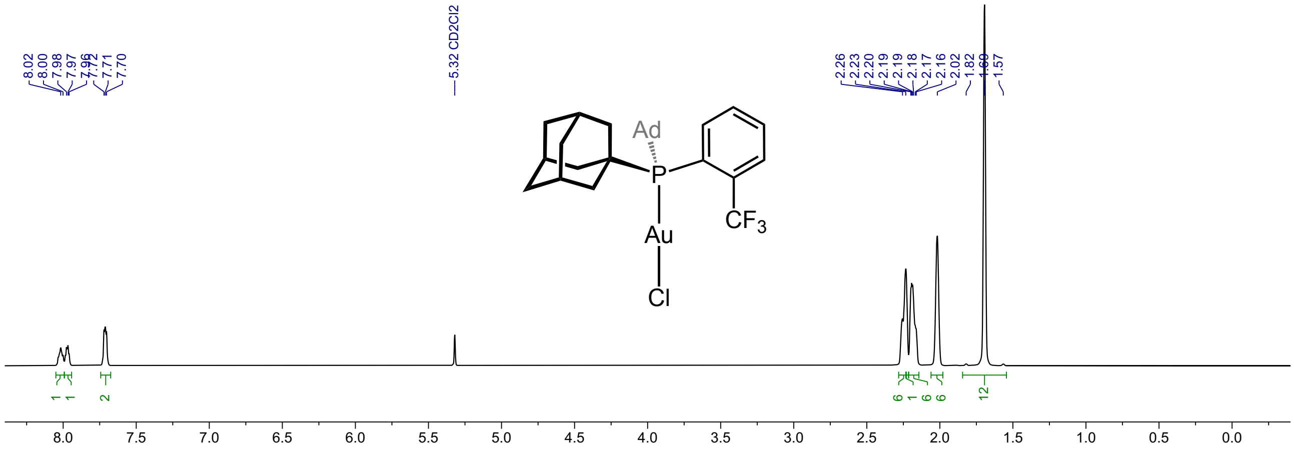

Figure S7. ^{1}H NMR spectrum of [Au(**L1**)Cl] in CD_2Cl_2 (500 MHz).

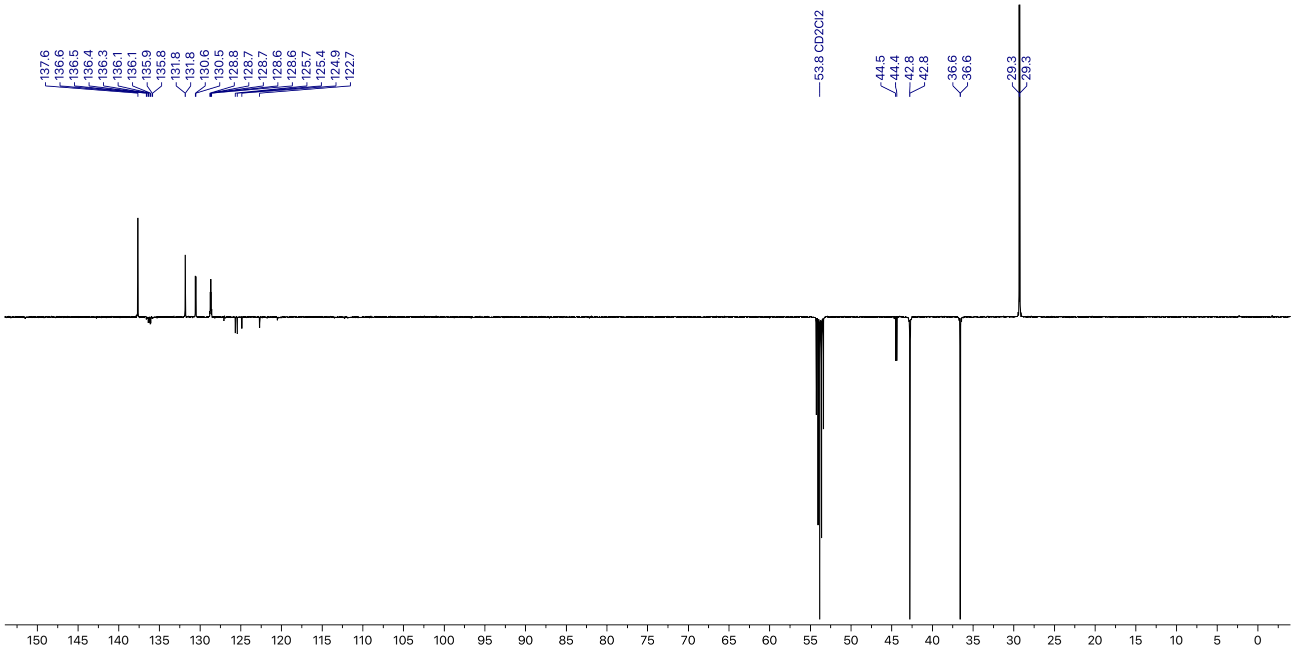

Figure S8. $^{13}C\{^{1}H\}$ APT NMR spectrum of [Au(**L1**)Cl] in CD_2Cl_2 (126 MHz).

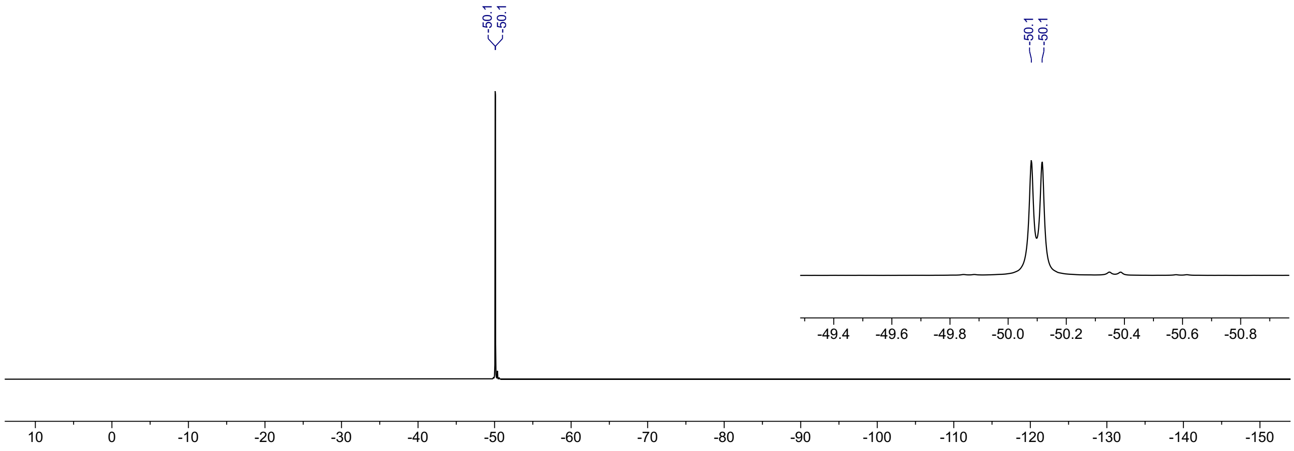

Figure S9. $^{19}F\{^{1}H\}$ NMR spectrum of [Au(**L1**)Cl] in CD_2Cl_2 (377 MHz).

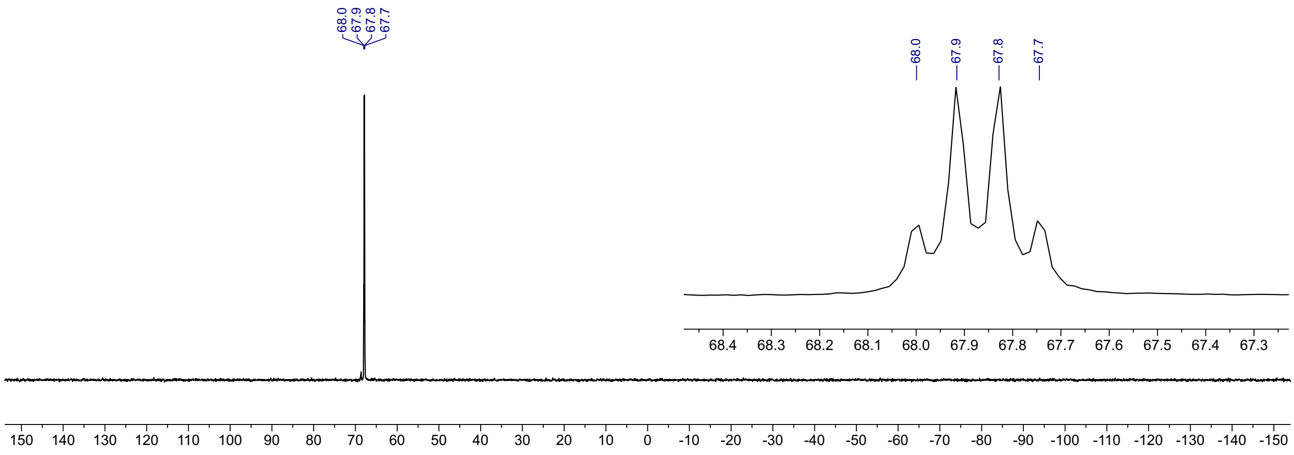

Figure S10. $^{31}P\{^{1}H\}$ NMR spectrum of [Au(**L1**)Cl] in CD_2Cl_2 (162 MHz).

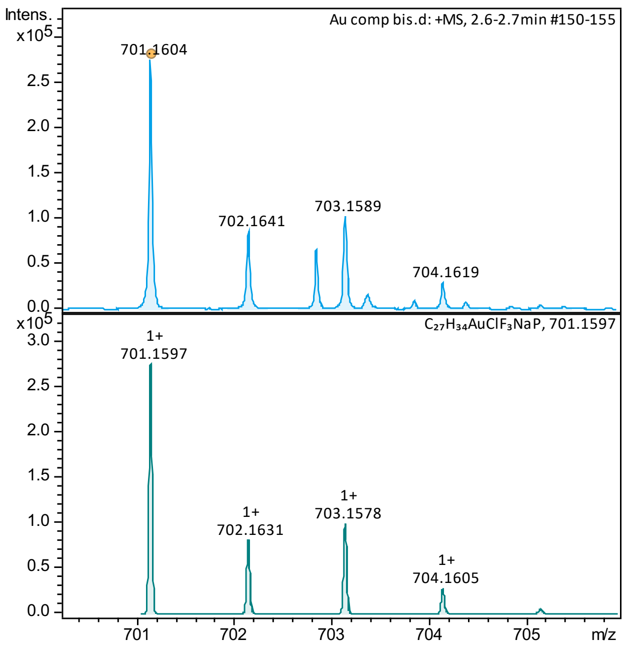

Figure S11. HR ESI-MS of [Au(**L1**)Cl].

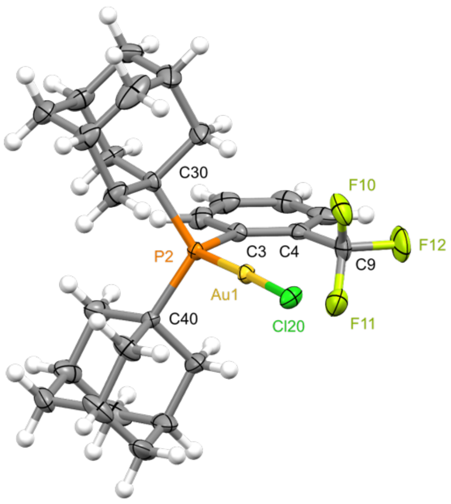

Figure S12. Solid-state structure of [Au(**L1**)Cl] with thermal ellipsoids at 50%; minor disordered component omitted for clarity. Selected metrics: Au1–P2, 2.254(2) Å; Au1–Cl20, 2.292(2) Å; Au1···F10, 2.986(6) Å; Au1···F11, 2.988(7) Å; P2–Au1–Cl20, 175.57(8)°; P2–C3–C4, 126.5(3)°; C3–C4–C9, 124.8(5)°; Au1–P2–C3–C4, 0.3(5)°; C3–C4–C9–F12, 178.5(7)°.

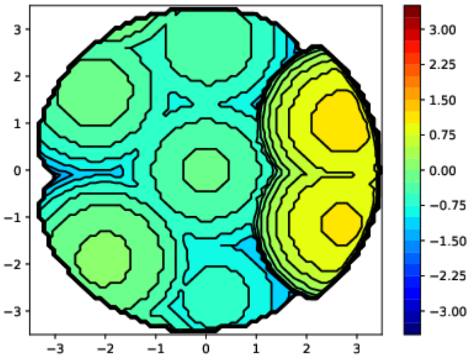

Figure S13. SambVca2 analysis of the steric profile of **L1** in [Au(**L1**)Cl]: $\%V_{bur}$ = 47.3%.

4 Preparation of [Au(L1)(OTf)]

A suspension of [Au(**L1**)Cl] (52.0 mg, 76.0 µmol) and AgOTf (21.6 mg, 84.0 µmol) in CH_2Cl_2 (5 mL) was stirred at room temperature in the dark for 2 h. The solution was filtered and reduced to dryness. The product was obtained as colourless crystals by recrystallisation from CH_2Cl_2/pentane at room temperature. Yield: 21.0 mg (26.5 µmol, 39%). Single crystals suitable for X-ray diffraction were obtained in this manner.

^{1}H NMR (CD_2Cl_2, 500 MHz): δ 7.97–8.04 (m, 2H, 3-Ar+6-Ar), 7.72–7.78 (m, 2H, 4-Ar+5-Ar), 2.24 (dm, $^2J_{HH}$ = 11.5, 6H, Ad{2-CH_2}), 2.15 (dm, $^2J_{HH}$ = 11.5, 6H, Ad{2-CH_2}), 2.04 (br, 6H, Ad{3-CH}), 1.70 (unresolved AB, 12H, Ad{4-CH_2}).
^{13}C{^{1}H} NMR (CD_2Cl_2, 126 MHz): δ 137.0 (d, $^2J_{PC}$ = 2, 6-Ar), 136.1 (qd, $^2J_{FC}$ = 30, $^2J_{PC}$ = 6, 2-Ar), 132.4 (d, $^4J_{PC}$ = 2, 4-Ar), 130.8 (d, $^3J_{PC}$ = 7, 5-Ar), 129.3 (app pent, J = 7, 3-Ar), 123.6 (d, $^1J_{PC}$ = 36, 1-Ar), 123.6 (q, $^1J_{FC}$ = 274, $PhCF_3$), 120.6 (q, $^1J_{FC}$ = 319, OTf), 45.1 (d, $^1J_{PC}$ = 23, Ad{1-C}), 42.9 (d, $^2J_{PC}$ = 2, Ad{2-CH_2}), 36.4 (d, $^4J_{PC}$ = 1, Ad{4-CH_2}), 29.3 (d, $^3J_{PC}$ = 10, Ad{3-CH}).
^{19}F{^{1}H} NMR (CD_2Cl_2, 377 MHz): δ −49.9 (d, $^{TS}J_{PF}$ = 13, 1F, $PhCF_3$), −77.5 (s, 1F, OTf).
^{31}P{^{1}H} NMR (CD_2Cl_2, 162 MHz): δ 68.0 (q, $^{TS}J_{PF}$ = 13).
HR ESI-MS (positive ion, 4 kV): 684.2276 ([*M*–OTf+NCMe]$^+$, calcd 684.2276) *m/z*.
Anal. calcd for $C_{28}H_{34}F_6O_3PSAu$ (792.57 g·mol^{-1}): C, 42.43; H, 4.32; N, 0.00. Found: C, 42.40; H, 4.34; N, 0.00.

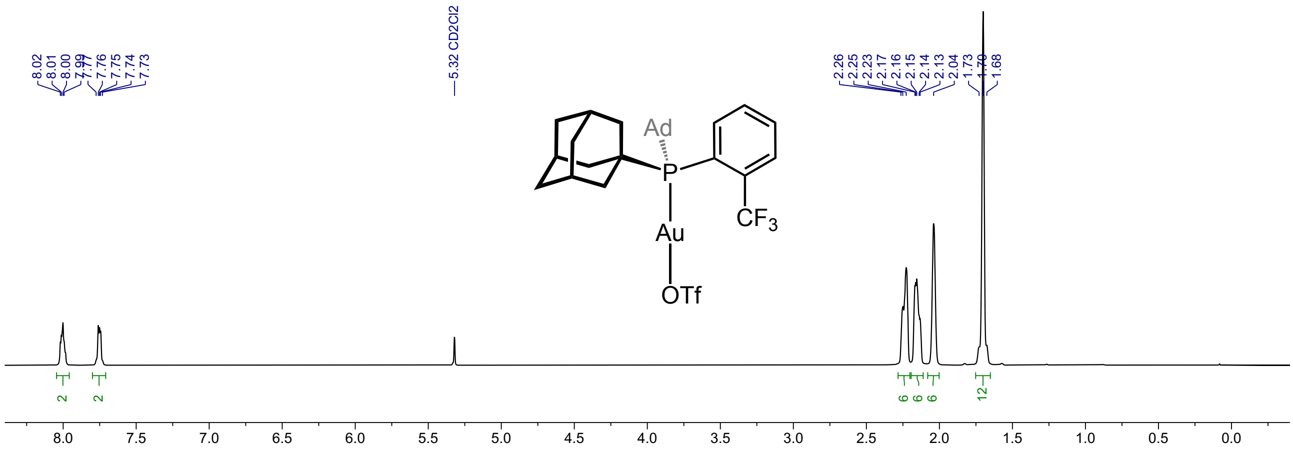

Figure S14. ^{1}H NMR spectrum of [Au(**L1**)(OTf)] in CD_2Cl_2 (500 MHz).

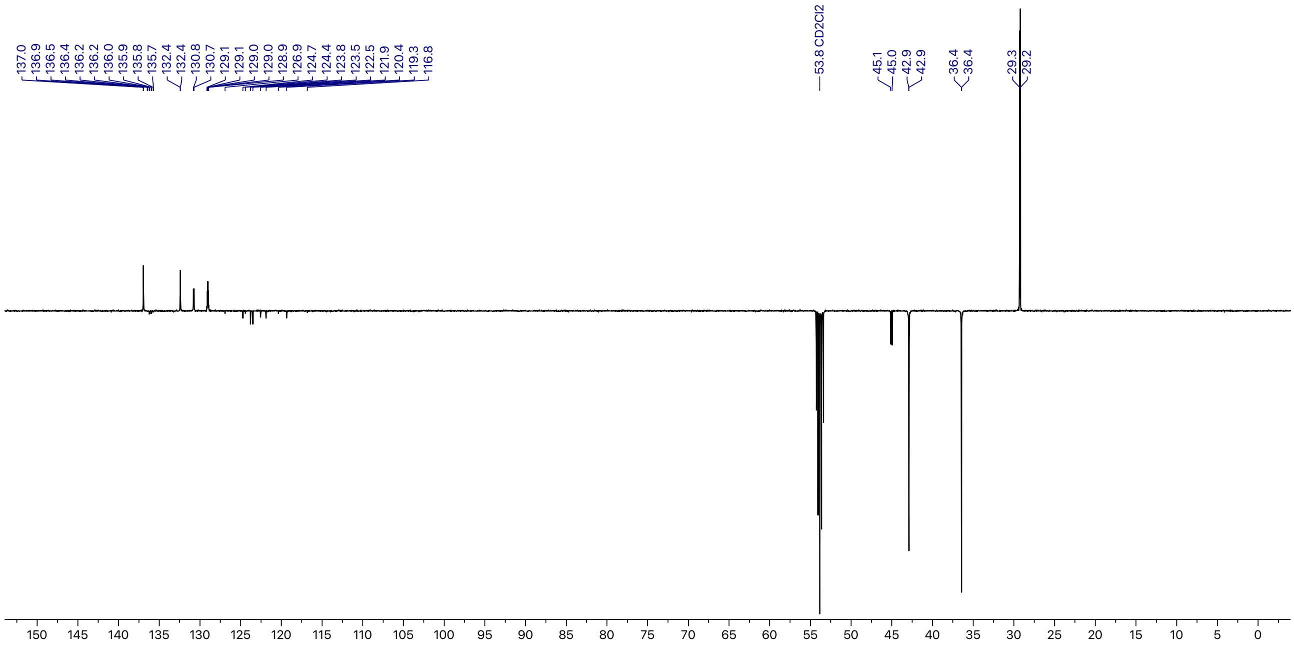

Figure S15. $^{13}C\{^{1}H\}$ APT NMR spectrum of [Au(**L1**)(OTf)] in CD_2Cl_2 (126 MHz).

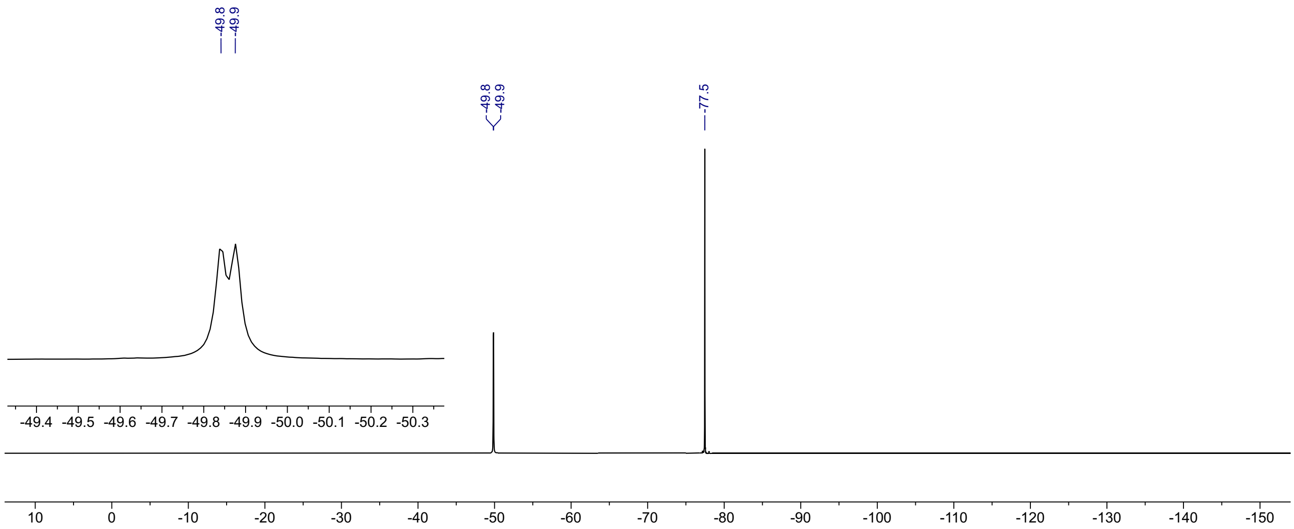

Figure S16. $^{19}F\{^{1}H\}$ NMR spectrum of [Au(**L1**)(OTf)] in CD_2Cl_2 (377 MHz).

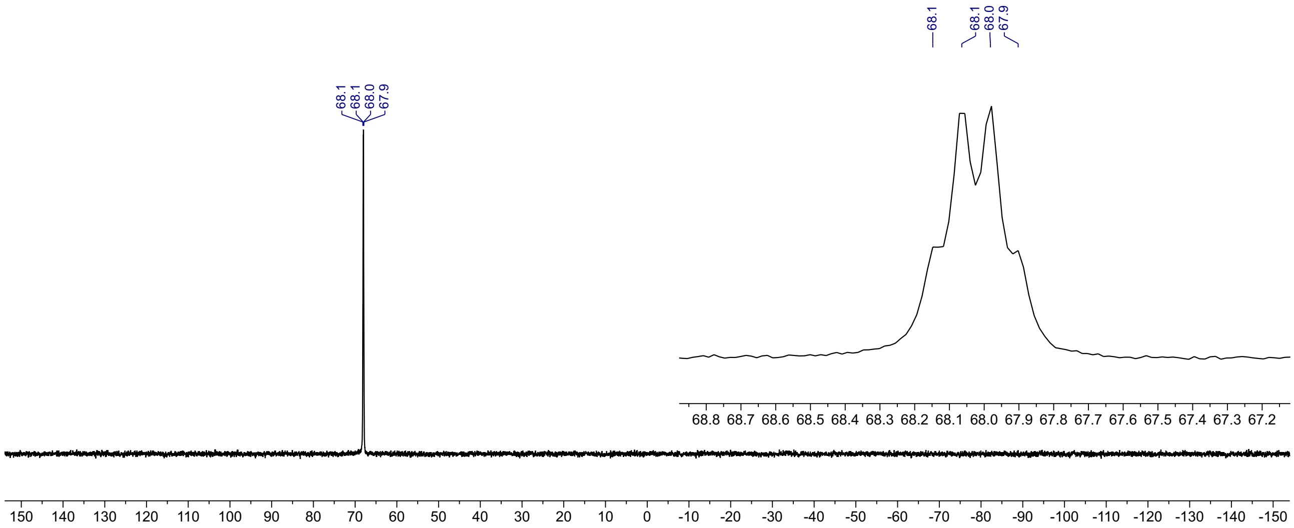

Figure S17. $^{31}P\{^{1}H\}$ NMR spectrum of [Au(**L1**)(OTf)] in CD_2Cl_2 (162 MHz).

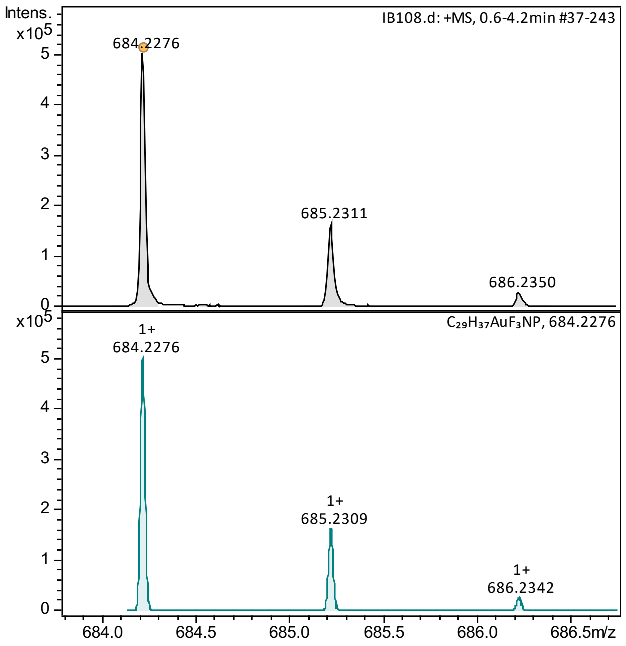

Figure S18. HR ESI-MS of [Au(**L1**)(OTf)].

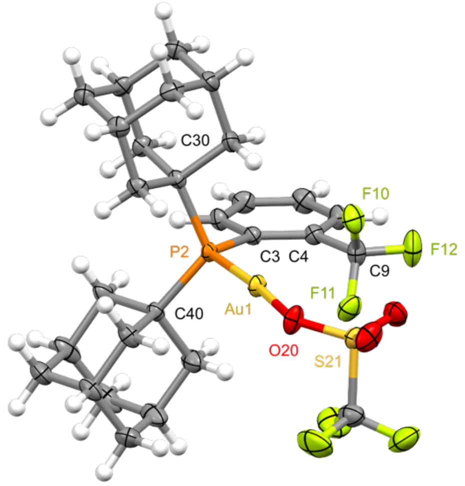

Figure S19. Solid-state structure of [Au(**L1**)(OTf)] with thermal ellipsoids at 50%. Selected metrics: Au1–P2, 2.2376(8) Å; Au1–O20, 2.089(2) Å; Au1···F10, 2.983(3) Å; Au1···F11, 2.967(2) Å; P2–Au1–O20, 174.03(7)°; P2–C3–C4, 128.9(2)°; C3–C4–C9, 124.0(3)°; Au1–P2–C3–C4, –8.6(3)°; C3–C4–C9–F12, 179.1(3)°.

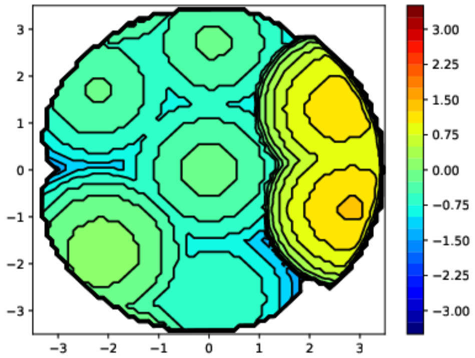

Figure S20. SambVca2 analysis of the steric profile of **L1** in [Au(**L1**)(OTf)]: %V_{bur} = 47.8%.

5 Steric profile of AdJohnPhos

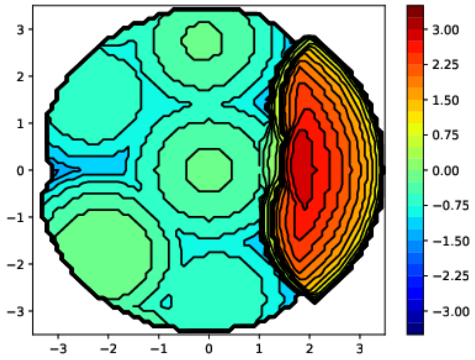

Figure S21. SambVca2 analysis of the steric profile of AdJohnPhos in [Au(AdJohnPhos)Cl] (CCDC 1550231): %V_{bur} = 51.4%.

6 Preparation of [Au(PAd_2Ph)Cl]

A solution of PAd_2Ph (37.8 mg, 100.0 µmol) and [Au(SMe_2)Cl] (29.5 mg, 100.0 µmol) in CH_2Cl_2 (5 mL) was stirred at room temperature for 3 h. Volatiles were removed *in vacuo* and the residue recrystallised from CH_2Cl_2/hexane at room temperature to afford the product as colourless crystals. Yield: 36.6 mg (59.9 µmol, 60%). Single crystals suitable for X-ray diffraction were obtained in this manner.

^{1}H NMR (CD_2Cl_2, 500 MHz): *δ* 8.17 (br, 1H, 2-Ph), 7.77 (br, 1H, 2-Ph), 7.56 (t, $^3J_{HH}$ = 7.1, 1H, 4-Ph), 7.50 (br, 2H, 2×3-Ph), 2.19 (dm, $^2J_{HH}$ = 10.5, 6H, Ad{2-CH_2}), 2.10 (dm, $^2J_{HH}$ = 10.5, 6H, Ad{2-CH_2}), 2.00 (br, 6H, Ad{3-CH}), 1.70 (unresolved AB, 12H, Ad{4-CH_2}).

^{13}C{^{1}H} NMR (CD_2Cl_2, 126 MHz): *δ* 142.2 (br, 2-Ph), 133.5 (br, 2-Ph), 132.0 (s, 4-Ph), 129.2 (br, 3-Ph), 128.3 (br, 3-Ph), 126.1 (d, $^1J_{PC}$ = 47, 1-Ph), 41.9 (d, $^2J_{PC}$ = 2, Ad{2-CH_2}), 41.3 (d, $^1J_{PC}$ = 25, Ad{1-C}), 36.6 (s, Ad{4-CH_2}), 29.0 (d, $^3J_{PC}$ = 10, Ad{3-CH}).

^{31}P{^{1}H} NMR (CD_2Cl_2, 162 MHz): *δ* 78.1.

HR ESI-MS (positive ion, 4 kV): 633.1712 ([*M*+Na]$^+$, calcd 633.1723) *m/z*.

Anal. calcd for $C_{26}H_{35}PAuCl$ (610.96 g·mol^{-1}): C, 51.11; H, 5.77; N, 0.00. Found: C, 50.86; H, 5.72; N, 0.00.

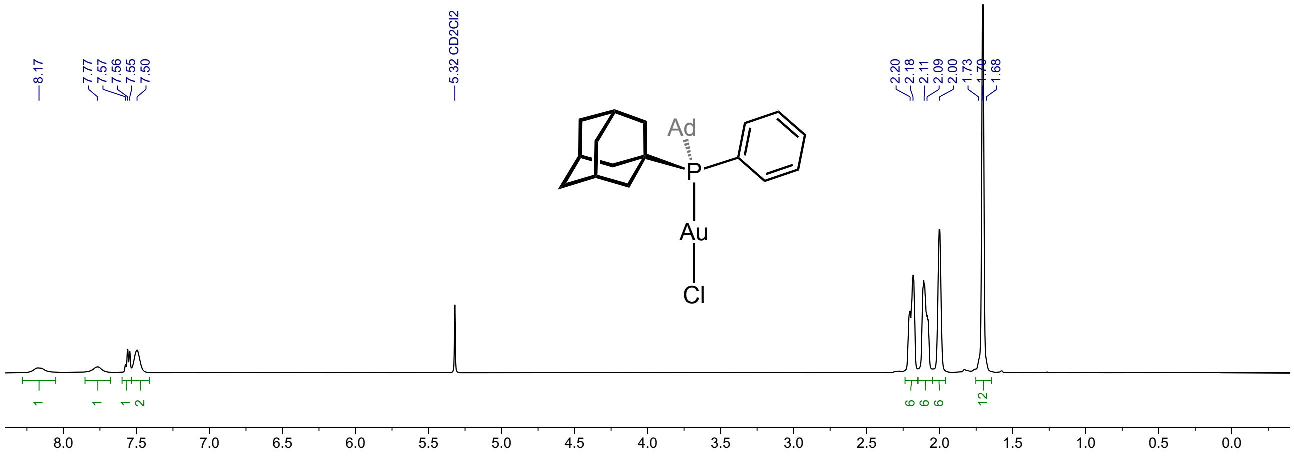

Figure S22. ^{1}H NMR spectrum of [Au(PAd_2Ph)Cl] in CD_2Cl_2 (500 MHz).

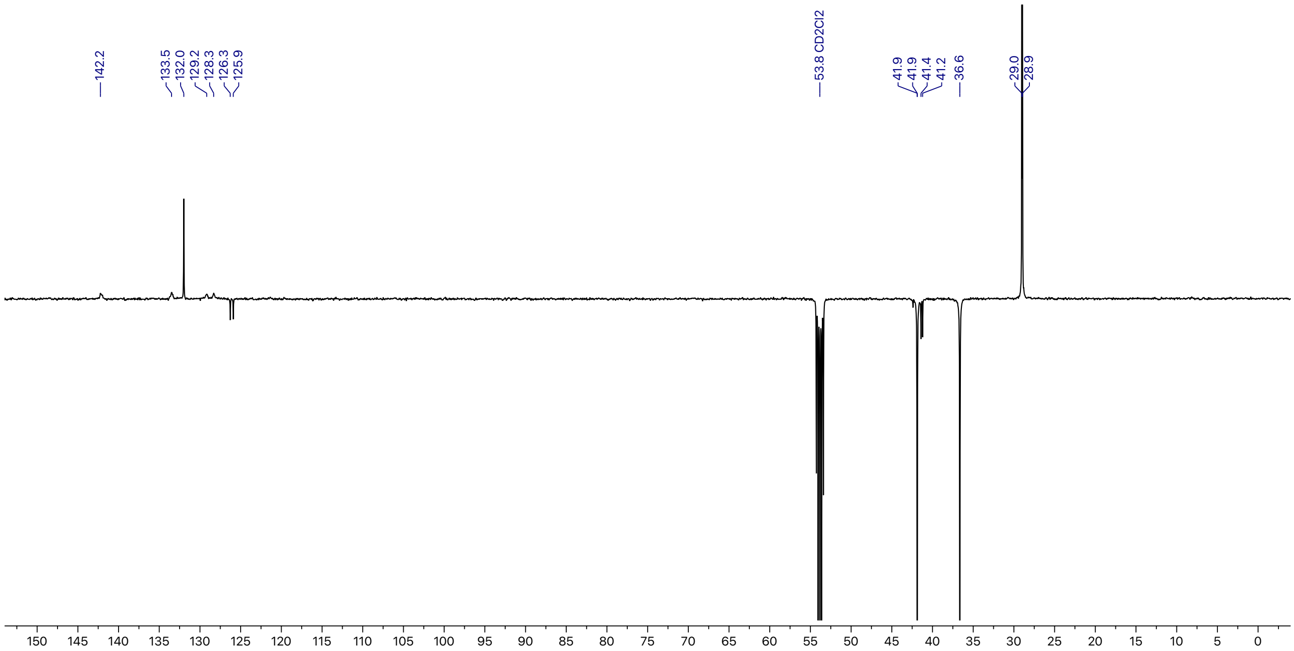

Figure S23. $^{13}C\{^{1}H\}$ APT NMR spectrum of [Au(PAd_2Ph)Cl] in CD_2Cl_2 (126 MHz).

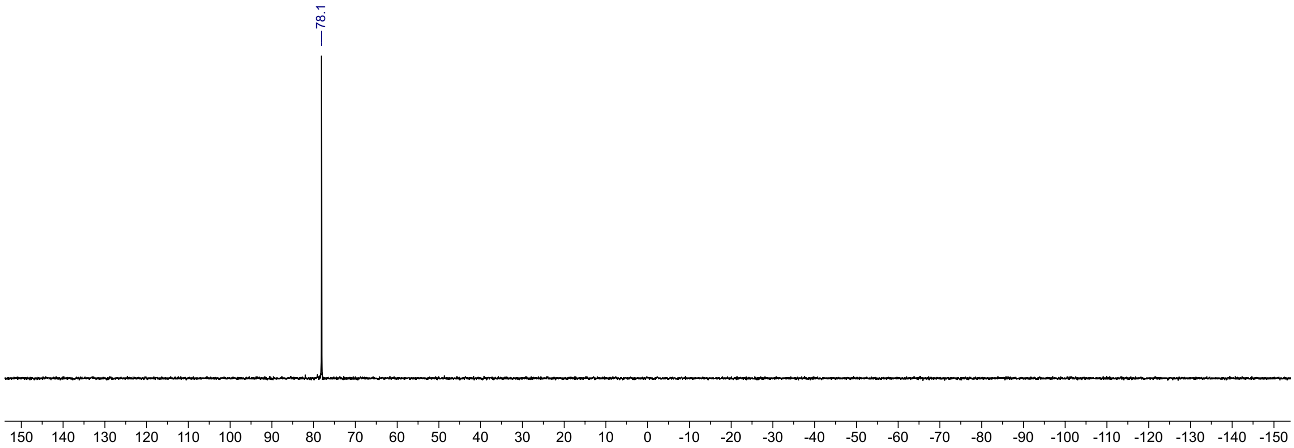

Figure S24. $^{31}P\{^{1}H\}$ NMR spectrum of [Au(PAd_2Ph)Cl] in CD_2Cl_2 (162 MHz).

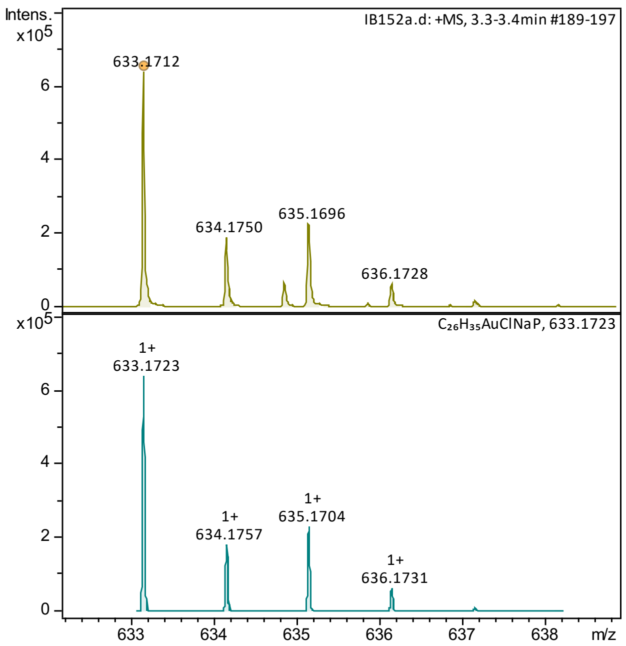

Figure S25. HR ESI-MS of [Au(PAd$_2$Ph)Cl].

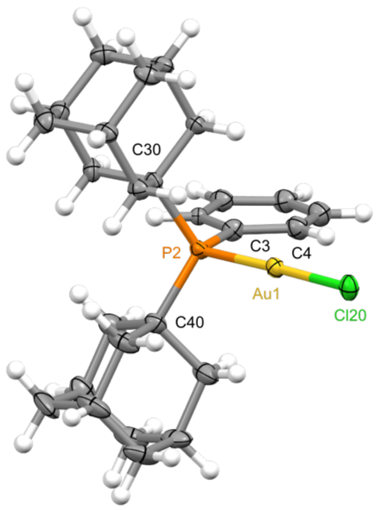

Figure S26. Solid-state structure of [Au(PAd$_2$Ph)Cl] with thermal ellipsoids at 30%; minor disordered component omitted for clarity. Selected metrics: Au1–P2, 2.294(3) Å; Au1–Cl20, 2.294(3) Å; P2–Au1–Cl20, 178.08(11)°; P2–C3–C4, 123.5(11)°; Au1–P2–C3–C4, 1.6(14)°;

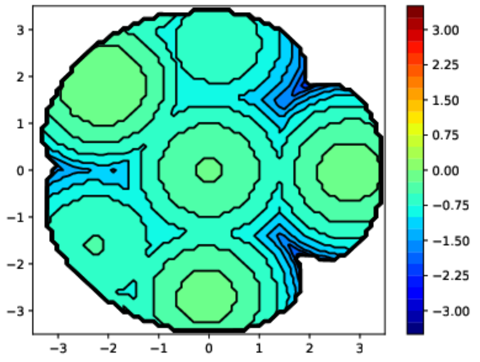

Figure S27. SambVca2 analysis of the steric profile of PAd$_2$Ph in [Au(PAd$_2$Ph)Cl]: %V_{bur} = 37.6%.

7 Catalysis data

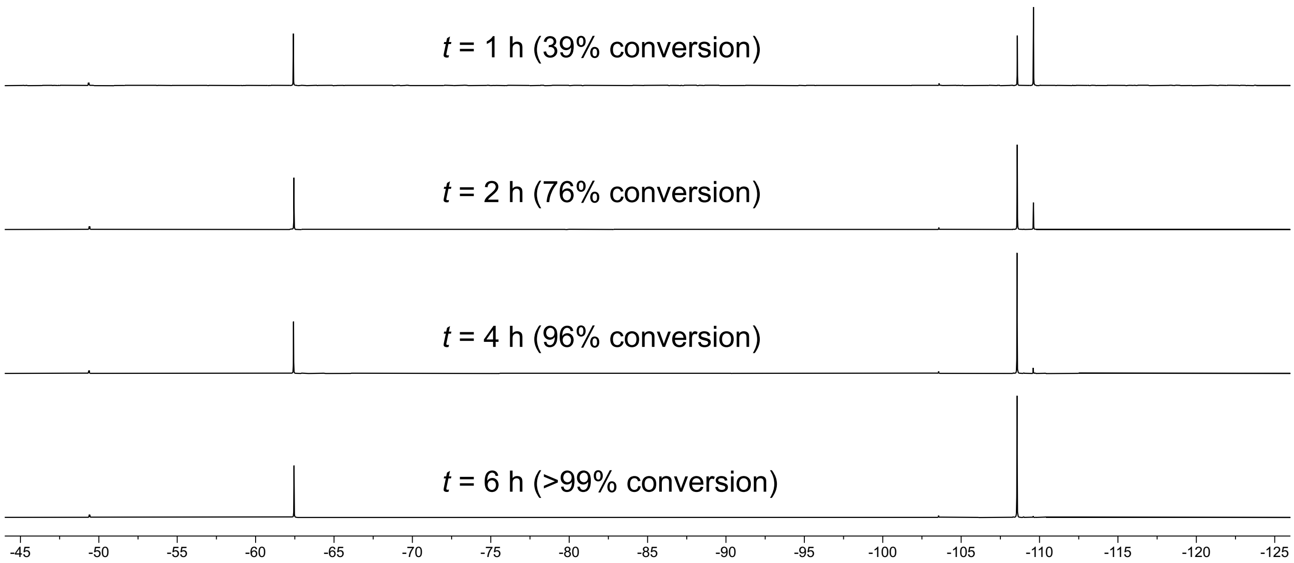

Figure S28. $^{19}F\{^1H\}$ NMR spectra collected during catalysis using [Au(**L1**)Cl]/Na[BAr$^F_4$] (377 MHz).

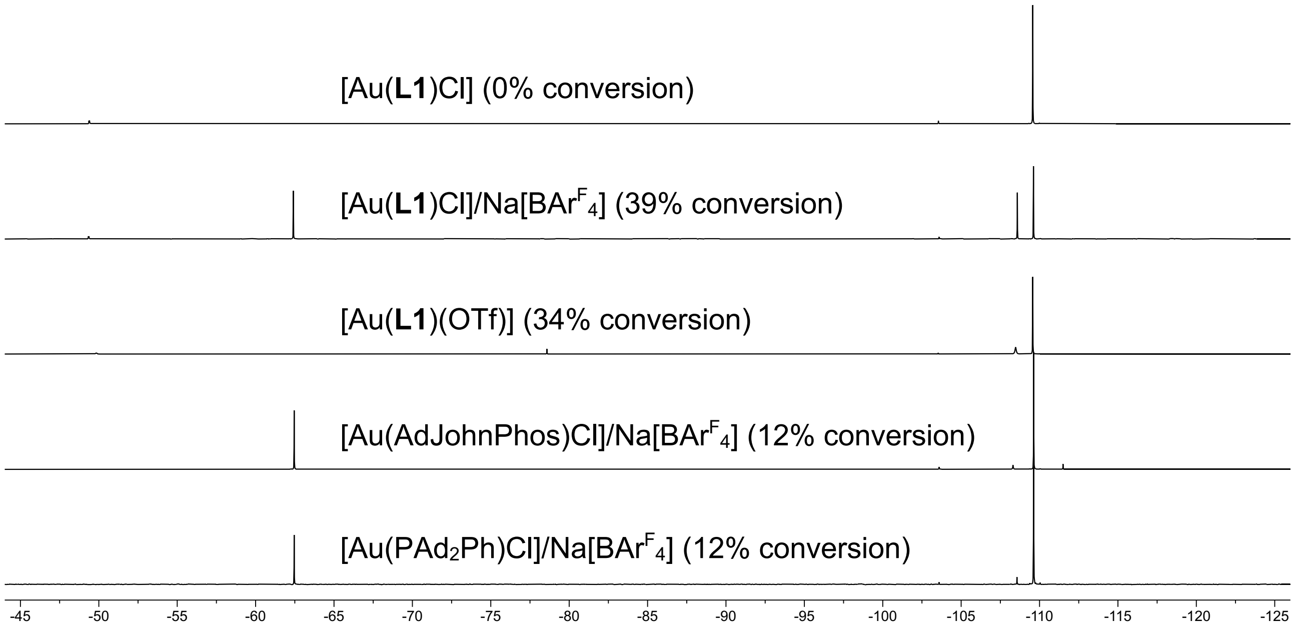

Figure S29. $^{19}F\{^1H\}$ NMR spectra collected during catalysis at *t* = 1 h (377 MHz).

8 References

[1] Barber, T.; Argent, S. P.; Ball, L. T. Expanding Ligand Space: Preparation, Characterization, and Synthetic Applications of Air-Stable, Odorless Di-*tert*-alkylphosphine Surrogates. *ACS Catal.* **2020**, *10*, 5454–5461.

[2] Köllhofer, A.; Plenio, H. Homogeneous Catalysts Supported on Soluble Polymers: Biphasic Sonogashira Coupling of Aryl Halides and Acetylenes Using MeOPEG-Bound Phosphine–Palladium Catalysts for Efficient Catalyst Recycling. *Chem. A Eur. J.* **2003**, *9*, 1416–1425.

[3] Abenante, L.; Quadros, G. T.; Perin, G.; Santi, C.; Penteado, F.; Lenardão, E. J. Visible-Light-Mediated Photocatalytic Synthesis of 2-Substituted Oxazole-5-carbaldehydes Promoted by Benzeneseleninic Acid. *Eur. J. Org. Chem.* **2022**, e202200641.

[4] (a) Martínez-Martínez, A. J.; Weller, A. S. Solvent-Free Anhydrous Li^+, Na^+ and K^+ Salts of $[B(3,5\text{-}(CF_3)_2C_6H_3)_4]^-$, $[BAr^F{}_4]^-$. Improved Synthesis and Solid-State Structures. *Dalton Trans.* **2019**, *48*, 3551–3554; (b) Buschmann, W. E.; Miller, J. S.; Bowman-James, K.; Miller, C. N. Synthesis of $[M^{II}(NCMe)_6]^{2+}$ (M = V, Cr, Mn, Fe, Co, Ni) Salts of Tetra[3,5-Bis(Trifluoromethyl)Phenyl]Borate. *Inorg. Synth.* **2002**, *33*, 83–91.

[5] Sheldrick, G. M. SHELXT – Integrated Space-Group and Crystal-Structure Determination. *Acta Cryst.* **2015**, *71*, 3–8.

[6] Dolomanov, O. V.; Bourhis, L. J.; Gildea, R. J.; Howard, J. A. K.; Puschmann, H. OLEX2: A Complete Structure Solution, Refinement and Analysis Program. *J. Appl. Cryst.* **2009**, *42*, 339–341.
